# Low prevalence of anti‐xenobiotic antibodies among the occupationally exposed individuals is associated with a high risk of cancer

**DOI:** 10.1002/cam4.1773

**Published:** 2018-12-21

**Authors:** Mohammad Sajid, Javed N. Agrewala

**Affiliations:** ^1^ Immunology Laboratory CSIR‐Institute of Microbial Technology Chandigarh India; ^2^ Indian Institute of Technology Ropar India

**Keywords:** anti‐xenobiotic antibodies, breast cancer, cancer, household contacts, pesticides, xenobiotics

## Abstract

Cancer is one of the major health problem globally, responsible for high morbidity and mortality. Exposure of humans to xenobiotics is associated with the development of cancer. Further, these xenobiotics may combine with the body proteins and can act as a hapten and elicit an antibody response. In this study, we examined whether the regular exposer to xenobiotics evokes anti‐xenobiotic antibodies and the presence of these antibodies have any correlation with the prevention of cancer. Interestingly, we noticed that the healthy household contacts showed significantly greater titers of anti‐xenobiotic antibodies, as compared to cancer patients. Consequently, suggesting that the higher level of anti‐xenobiotic antibodies may be responsible for neutralizing the effect of xenobiotics in the healthy subjects. Thereby, preventing the individuals from disease. In contrast, the presence of a significantly lower level of anti‐xenobiotic antibodies in the cancer patients may be a causative factor for disease infliction. In conclusion, immunotherapy employing anti‐xenobiotic antibodies may provide a prudent remedial measure to clear xenobiotics from the body of the individuals and thereby protecting from cancer.

## 1 INTRODUCTION

According to World Health Organization report, cancer is a major health problem worldwide and second leading cause of casualties (8.8 million) during 2015.^1^ The factors responsible for cancer are oncogenic viruses, ionizing radiations, and environmental pollutants, such as xenobiotics. Xenobiotics exposure causes numerous medical problems such as Parkinson's disease, diabetes, autoimmune disorders, and cancer.^2^, ^3^, ^4^, ^5^ Epidemiological and animal studies have established the role of pesticides, polycyclic aromatic hydrocarbons, mycotoxins, and heavy metals in causing cancer. It has been reported that the interaction of these xenobiotics with self‐molecules, such as DNA, proteins, and lipids, alters various cellular processes. The genetic and epigenetic changes in the cellular processes can instigate cancer.^6^

Majority of xenobiotics are detoxified and excreted out from the body. Liver is the central organ to metabolize xenobiotics. Xenobiotics can be converted into their reactive derivatives by the action of various metabolizing enzymes, such as cytochromes‐P450, glutathione‐S‐transferases, and glucuronosyl transferases, etc.^7^ Other organs such as kidney, lung, and skin provide a site for xenobiotics exposure and metabolism. Further, innate immune cells such as monocytes, macrophages, dendritic cells, and polymorphonuclear cells play a fundamental role in xenobiotics metabolism. The metabolic conversion of xenobiotics was observed in dermal Langerhans cells (immature dendritic cells), which contains the cytochrome P4501A (CYP1A) enzyme. After the conversion by CYP1A enzyme, xenobiotics form a complex with self‐proteins, which are processed and presented in context with MHC class‐I and MHC class‐II molecules.^8^ The xenobiotic‐protein complex presented by antigen presenting cells (APCs) is subsequently recognized by T cells, which provides help to B cells for antibody (Ab) production.^9^, ^10^

The conjugation of xenobiotics to self‐proteins makes them highly immunogenic and therefore elicits the production of anti‐xenobiotic Abs.^11^, ^12^, ^13^, ^14^ Generation of anti‐xenobiotic Abs have a physiological role in the clearance of xenobiotics from the body. The anti‐xenobiotic Abs bind to xenobiotics, and these antibodies can block cancer provoking property of xenobiotics. Formation of immune complexes between xenobiotics and anti‐xenobiotic Abs helps in increasing the clearance of xenobiotics from the body.^15^

Punjab is a leading state of India for agricultural farming. The farmers in this area use pesticides extensively. Cancer prevalence in the people of this region is significantly higher, as compared to other parts of the country. Based on the above‐mentioned studies, we propose that the individuals that are regularly exposed to xenobiotics may elicit the generation of anti‐xenobiotic Abs in their body. Further, we want to test whether these Abs play any fundamental role in preventing or provoking cancer. Interestingly, we observed a high titer of anti‐xenobiotic Abs in the serum of healthy household contacts. In contrast, low level of Abs was detected in the cancer patients. Thus, this study illustrates that the high titer of anti‐xenobiotic Abs in healthy subjects may play a role in protection against cancer.

## 2 MATERIALS AND METHODS

### 2.1 Chemicals

Bovine serum albumin (BSA), rabbit serum albumin (RSA), ovalbumin (OVA), 3,4‐dichloroaniline, atrazine desethyl (6‐Chloro‐N,N‐dimethyl‐1,3,5‐triazine‐2,4‐diamine), N‐(1‐naphthyl)‐ethylene‐diamine, 2‐benzimidazole propionic acid, aflatoxin‐BSA conjugate (hapten density of 8‐12), N‐hydroxysuccinimide (NHS) ester, O‐phenylenediamine dihydrochloride (OPD), 2‐(N‐morpholino) ethanesulfonic acid (MES), and dimethylformamide were purchased from Sigma‐Aldrich (St. Louis, MO). Goat anti‐human IgG+IgM+IgA‐HRP labeled Abs were purchased from Abcam (Cambridge, MA). Vacutainer tubes were obtained from BD Diagnostics (Franklin Lakes, NJ). EDC [1‐ethyl‐3‐(3‐dimethyl aminopropyl) carbodiimide] and Nunc maxiSorp flat‐bottom plates were purchased from Thermo Fisher Scientific (Waltham, MA). All the reagents and solvents were of analytical grade.

### 2.2 Study population and ethical clearance

Blood samples of 75 cancer patients and 30 healthy household contacts were acquired from the Advanced Cancer Centre, Guru Govind Singh Medical College, Faridkot, Punjab, India. The study population includes agricultural farmers involved in the cultivation and harvesting of crops. These farmers handled, sprayed, and stored various pesticides for crops protection. The written informed consent was obtained from the participants of this study. Ethical clearance was approved by the Department of Family and Health, Punjab, India (No. 5/59/13‐HB4/1891). The blood samples (4‐5 mL) were collected in EDTA‐coated serum separator vacutainer tubes. The blood containing tubes were centrifuged at 847 × g for 20 minutes, and the serum was separated and frozen (−80°C), until subsequent analysis. The details of cancer patients and healthy individuals are provided in Table S1. The use of toxic chemicals was approved by the Institutional Biosafety Committee of the CSIR‐Institute of Microbial Technology, Chandigarh, India (IMTECH/IBSC/2013/5).

### 2.3 Conjugation of xenobiotics to the carrier proteins

The xenobiotic molecules having a carboxylic (–COOH) and amine (‐NH_2_) groups were used for conjugation with the protein molecules or *vice versa*. Briefly, bovine serum albumin (BSA) (0.15 mmol/L) in MES buffer (100 mmol/L, pH 4.5) was mixed with 1‐ethyl‐3‐(3‐dimethyl aminopropyl) carbodiimide (EDC) (75 μmol/L) and N‐hydroxysuccinimide (NHS) (75 μmol/L). The mixture was incubated for 15 minutes at room temperature followed by centrifugation at 15160 ×g for 20 minutes at 4°C. The supernatant was collected and further mixed with different molar ratio of xenobiotics prepared in dimethylformamide, and the volume was raised to 1 mL using borate buffer (50 mmol/L, pH 9.2). The mixtures were incubated overnight at 4°C on a rotator. Different mixtures of xenobiotic‐BSA conjugates were centrifuged at 15160 ×g for 20 minutes at 4°C. The supernatants were collected and dialyzed against phosphate buffer saline (PBS) for 40 hours with a change of buffer three times. The conjugates were stored in the deep freezer (−80°C) till subsequent use. Similar to BSA, dichloroaniline was conjugated to RSA and OVA, as described above. The emission spectra were recorded at a scan rate of 5 nm/s using a fluorescence spectrophotometer (Varian Cary Eclipse, Santa Clara, CA). Further, MALDI‐TOF spectroscopy was performed for mass determination of all conjugates.

### 2.4 Determination of specificity of Ab against xenobiotics

Sera of 8 different individuals that were tested positive for anti‐diuron Abs were monitored for the presence of Abs against diuron conjugated to carrier proteins BSA, RSA and OVA. Briefly, 96‐well ELISA plates (Nunc maxiSorp flat bottom) were coated with 100 μL of 10 μg/mL of diuron‐BSA, diuron‐RSA, and diuron‐OVA prepared in carbonate/bicarbonate buffer (200 mmol/L, pH 9.6). The plates were incubated overnight at 4°C. The plates were then washed three times with PBS containing 0.05% Tween‐20 (PBST‐20). The unbound sites were blocked (200 μL/well) with defatted skimmed milk (5.0%) in PBS for 2 hours at 37°C. The plates were subsequently washed thoroughly with PBST‐20 (4 times). Different dilutions of serum samples were prepared in PBST‐20+ defatted skimmed milk (2%) solution to avoid cross‐reactivity. The serum dilutions were added (50 μL/well) to the respective wells and incubated overnight at 4°C. The plates were washed five times with PBST‐20. The secondary Ab, goat anti‐human IgG+IgM+IgA labeled with HRP (horseradish peroxidase) was prepared in PBST‐20+ defatted skimmed milk (2%) solution and added to the wells (100 μL/well) with appropriate dilution (1:5000). All dilutions were thoroughly standardized beforehand. The plates were then incubated for 45 minutes at 37°C followed by washing with PBST‐20 six times. Finally, for color development, OPD (10 mg/10 mL) and 30% H_2_O_2_ (10 μL/10 mL) prepared in citrate buffer (pH 5) was added (100 μL/well) to each well and incubated for 15 minutes. The reaction was stopped by adding 1 mol/L H_2_SO_4_ (50 μL/well), and absorbance was measured at 490 nm using ELISA reader (Synergy, Biotek, Mumbai, India). To identify nonspecific bindings, several control wells with all reagents except human serum were used in each assay. To establish the specificity of the anti‐xenobiotic Abs, the control wells were coated with carrier only proteins BSA, RSA, and OVA. The control wells were also coated with carbonate/bicarbonate buffer. Correspondingly, antibody response against different xenobiotics, *viz*. atrazine, carbendazim, naphthalene, and aflatoxin was determined in the serum of cancer patients and healthy household contacts by ELISA, as described above.

### 2.5 Serial dilution assay and measurement of Ab titer

The specificity of anti‐xenobiotic Abs was determined by serial dilutions of serum samples (log_10_ dilution). The serial dilution curve was made after completion of the ELISA assay to determine the titer of Abs for subsequent analysis of all the serum samples. In order to obtain the Abs response against the xenobiotics alone and not the carrier protein, the background of the Ab response obtained against the carrier BSA protein was subtracted from the xenobiotics‐BSA conjugates. For Ab titer calculation against the xenobiotics, 0.2 optical density (OD) was used as a cutoff (to overcome background). The titer against specific xenobiotics was calculated by plotting Ab dilutions on a log scale (*x*‐axis) against the OD in a linear scale (*y*‐axis). The Ab titer is the dilution factor of the serum at which the OD of the sample is equal to the cut‐off OD.

### 2.6 Statistical analysis

The data are represented as the mean ± standard error. The statistical analysis was performed using nonparametric test Mann‐Whitney *U* test to evaluate the significance of differences between a pair of data sets. A *P*‐value of ≤0.05 was considered to be statistically significant.

## 3 RESULTS

### 3.1 Conjugation of xenobiotics to BSA causes quenching of tryptophan fluorescence of BSA

**Figure 1:**
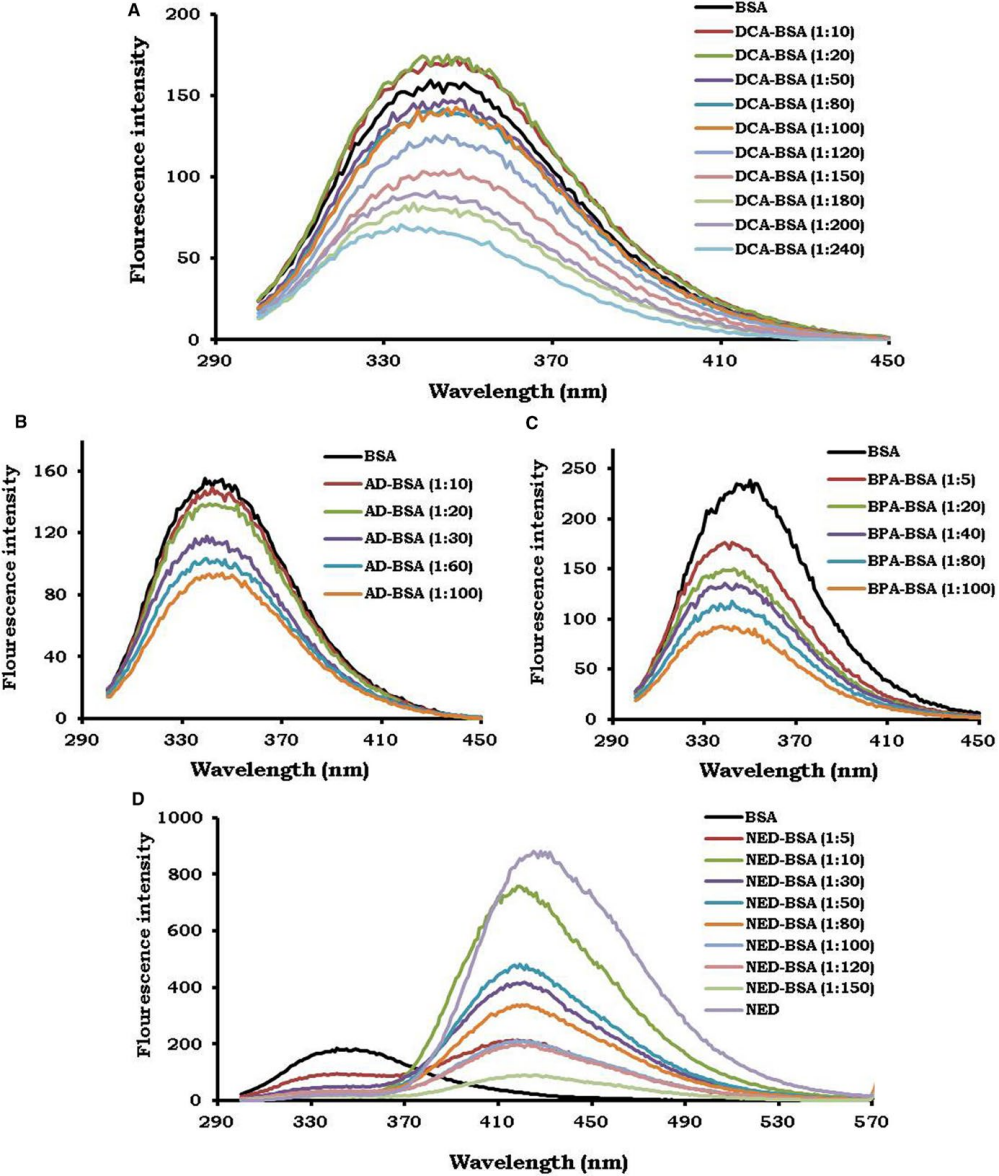
Conjugation of xenobiotics causes quenching of tryptophan fluorescence of BSA. The xenobiotic molecules were conjugated to BSA with increased molar ratios. The fluorescence emission spectra (300‐570 nm) of xenobiotic‐BSA conjugates were measured after excitation at 290 nm. The fluorescence emission spectra of BSA was measured for each BSA‐xenobiotic conjugates, as indicated in the figures for the: (A) dichloroaniline (DCA); (B) atrazine desethyl (AD); (C) benzimidazole propionic acid (BPA); (D) naphthylethylenediamine (NED). The fluorescence of NED alone (10 μg/mL) was depicted as a violet line. The fluorescence of BSA was taken as a control (black line)

We performed fluorescence spectroscopy to determine the spectra of xenobiotic‐BSA conjugates. The change in tryptophan fluorescence intensity was used to monitor ligand–protein interaction.^16^ For characterization of xenobiotic‐BSA conjugates, tryptophan fluorescence emission spectra were taken after excitation at 290 nm.^17^ A novel derivative of diuron, that is, dichloroaniline (DCA) was conjugated to BSA with increasing molar ratios (Figure 1A). The emission intensity of tryptophan of BSA was progressively quenched, with the increase in the molar concentration of DCA molecules. There was a significant decrease in the emission maxima of tryptophan residues of DCA‐BSA, as compared to BSA (Figure 1A). Maximum inhibition of fluorescence intensity of tryptophan residues caused by DCA attached to BSA was observed at a ratio of 1:240. Consequently, a decrease in the fluorescence emission of tryptophan residues of BSA denoted that DCA molecules were attached to BSA. Therefore, the increment in the concentration of DCA signifies more number of DCA coupled to BSA (Figure 1A). Similarly, a derivative of atrazine known as atrazine desethyl (AD) was conjugated to BSA with the different molar ratios (Figure 1B). BSA showed maximum fluorescence intensity at 340 nm. However, the emission maxima of tryptophan fluorescence were found to be progressively quenched with an increase in the number of AD linked to BSA. The maximum inhibition of fluorescence intensity of tryptophan residue caused by AD bonding to BSA was observed at a ratio 1:100 (Figure 1B). The decrease in the fluorescence intensity of tryptophan residues was due to the linkage of the AD to BSA. The successive decline in the fluorescence intensity corresponds to enhancement in the conjugation frequency of AD to BSA (Figure 1B). Likewise, a carbendazim derivative, that is, benzimidazole propionic acid (BPA) was conjugated to BSA at various molar ratios (Figure 1C). The major decrease in the fluorescence intensity of tryptophan fluorescence of BSA was noticed due to attachment of BPA molecules to BSA. The maximum inhibition of fluorescence intensity of tryptophan residue caused by BPA bonding to BSA was observed at a ratio of 1:100 (Figure 1C). The fluorescence intensity of BSA tryptophan moiety was progressively inhibited due to an increase in the number of BPA molecules attached to BSA. Lastly, a derivative of naphthalene, that is, N‐(1‐naphthyl)‐ethylenediamine (NED) was conjugated to BSA at different molar ratios (Figure 1D). The fluorescence of BSA showed emission maxima at 340 nm. However, it was observed that the fluorescence intensity of BSA was gradually reduced with the increase in the molar ratio of NED (Figure 1D). Another emission spectrum was observed in a range of 350‐570 nm, which matched with the emission spectra of NED (Figure 1D). Therefore, emission observed by NED‐BSA conjugates in the range of 350‐570 nm established that NED was conjugated to BSA. Consequently, we observed that the emission intensity of tryptophan residues of BSA was progressively quenched with an increase in the molar ratio of xenobiotic molecules. The decrease in the fluorescence emission of tryptophan residues of BSA denoted that xenobiotics were attached to BSA. Therefore, the increment in the concentration of xenobiotics signifies enhancement in the number of xenobiotics coupled to BSA (Figure 1A‐D).

The level of conjugation was determined by observing the molecular mass of each BSA‐xenobiotics conjugate compared to BSA by mass spectroscopy. Two peaks were observed in the mass spectrum, which represents states of charge on protein due to the ionization. One state was singly charged (M+H)^+^ while other was doubly charged (M+2H)^++^. The actual molecular mass of BSA and each BSA‐xenobiotic conjugate was represented by a centroid of doubly charged state peak, that is, (M+2H)^++^, as determined by another study.^18^ The increase in the concentration of xenobiotic leads to an augmentation in the molecular mass of BSA‐xenobiotic conjugates. This signifies a linkage between BSA and number of the xenobiotic molecules. The DCA molecules were conjugated to BSA, as evidenced by an increase in the molecular mass of DCA‐BSA (Figure S1). A maximum number of DCA molecules (~16) conjugated to BSA was found at 1:240 (Table S2). The ratios 1:180 and 1:200 showed approximately 9 and 12 molecules of DCA were attached to BSA, respectively (Table S2). The conjugation of the AD to BSA resulted in an expansion in the molecular mass of AD‐BSA conjugates. The increased mass of each AD‐BSA conjugate was observed by a peak (doubly charged) obtained in the mass spectra (Figure S2). The difference in the molecular mass due to the incorporation of the AD to BSA corresponds to the number of AD molecules coupled to a single BSA. A significant number of AD molecules (~45) were conjugated per BSA molecule at a ratio 1:100 (Table S3). Further, we observed that the molecular mass of each BPA‐BSA conjugate was increased due to attachment of BPA to BSA (Figure S3). The higher molar ratio of BPA resulted in a remarkable increase in the molecular mass of BPA‐BSA conjugates. The molecular mass analysis showed a maximum number (~60) of BPA molecules conjugated to each BSA at a ratio 1:100 (Table S4). Furthermore, the intact mass of NED‐BSA was determined by mass spectroscopy. The conjugate density was determined by observing the improvement in a molecular mass of NED‐BSA compared to BSA (Figure S4). A maximum number of NED molecules (~14) conjugated to each BSA molecule was found to be at a ratio 1:100 (Table S5). Therefore, an enhancement in the molecular mass corresponds to the increase in the number of xenobiotic molecules per BSA.

### 3.2 Conjugation of dichloroaniline causes quenching of fluorescence of tryptophan residue of RSA and OVA

**Figure 2:**
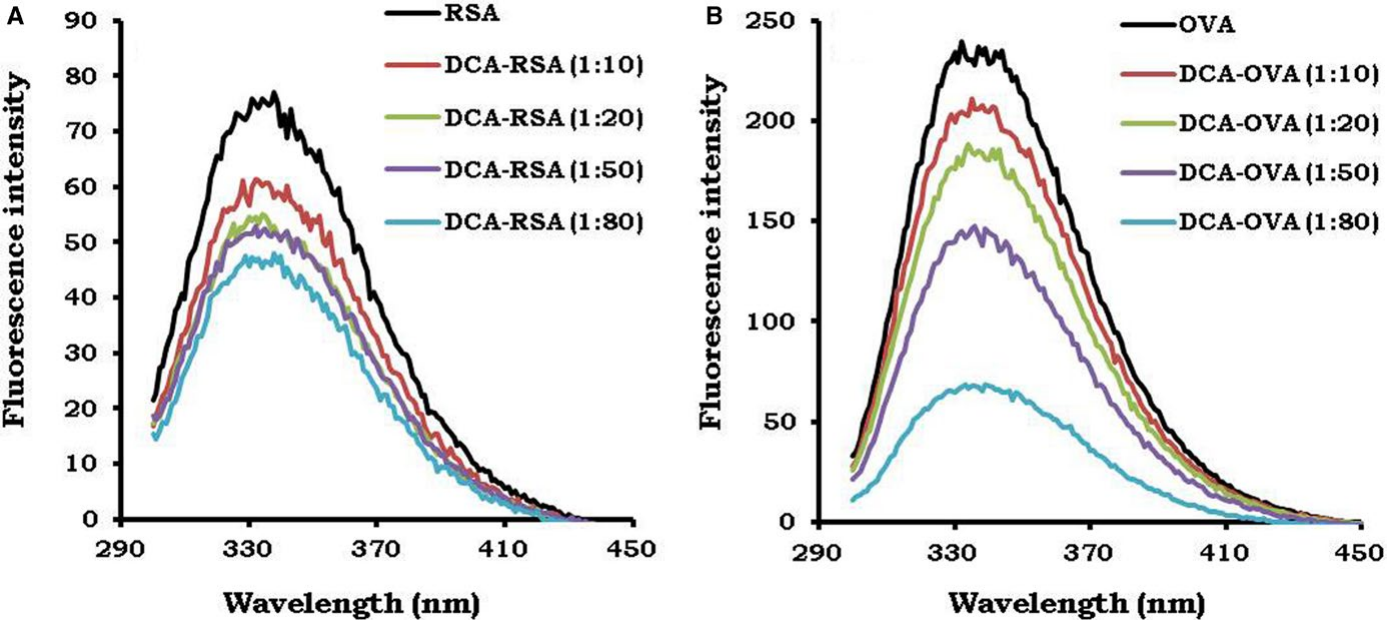
Conjugation of dichloroaniline (DCA) causes quenching of fluorescence of tryptophan residue of RSA and OVA. Different molar ratios: 1:10 to 1:80 of DCA were used to conjugate with RSA and OVA. The fluorescence emission spectra (300‐450 nm) of (A) RSA‐DCA; (B) OVA‐DCA conjugates were measured after excitation at 290 nm. The fluorescence of RSA and OVA alone was taken as a control (black line)

Like BSA, DCA was conjugated to RSA and OVA at an increased molar ratio (Figure 2). The fluorescence emission spectra of DCA‐RSA and DCA‐OVA were recorded in the range of 300‐450 nm, after excitation at 290 nm. The quenching of fluorescence intensity of RSA and OVA designated their conjugation with DCA (Figure 2A,B). There was a gradual reduction in the fluorescence intensity of RSA and OVA with an increase in the concentration of DCA. The fluorescence spectra of RSA and OVA were taken as controls (Figure 2A,B). Further, the molecular mass of DCA‐RSA and DCA‐OVA was determined by mass spectroscopy. The mass spectra analysis determined the extent of conjugation of DCA to RSA and OVA (Figures S5 and S6). The maximum number of DCA molecules conjugated to RSA and OVA were 8 and 5, respectively (Tables S6 and S7).

### 3.3 Antibody response observed was specific to xenobiotic, irrespective of its conjugation with different carrier proteins

**Figure 3:**
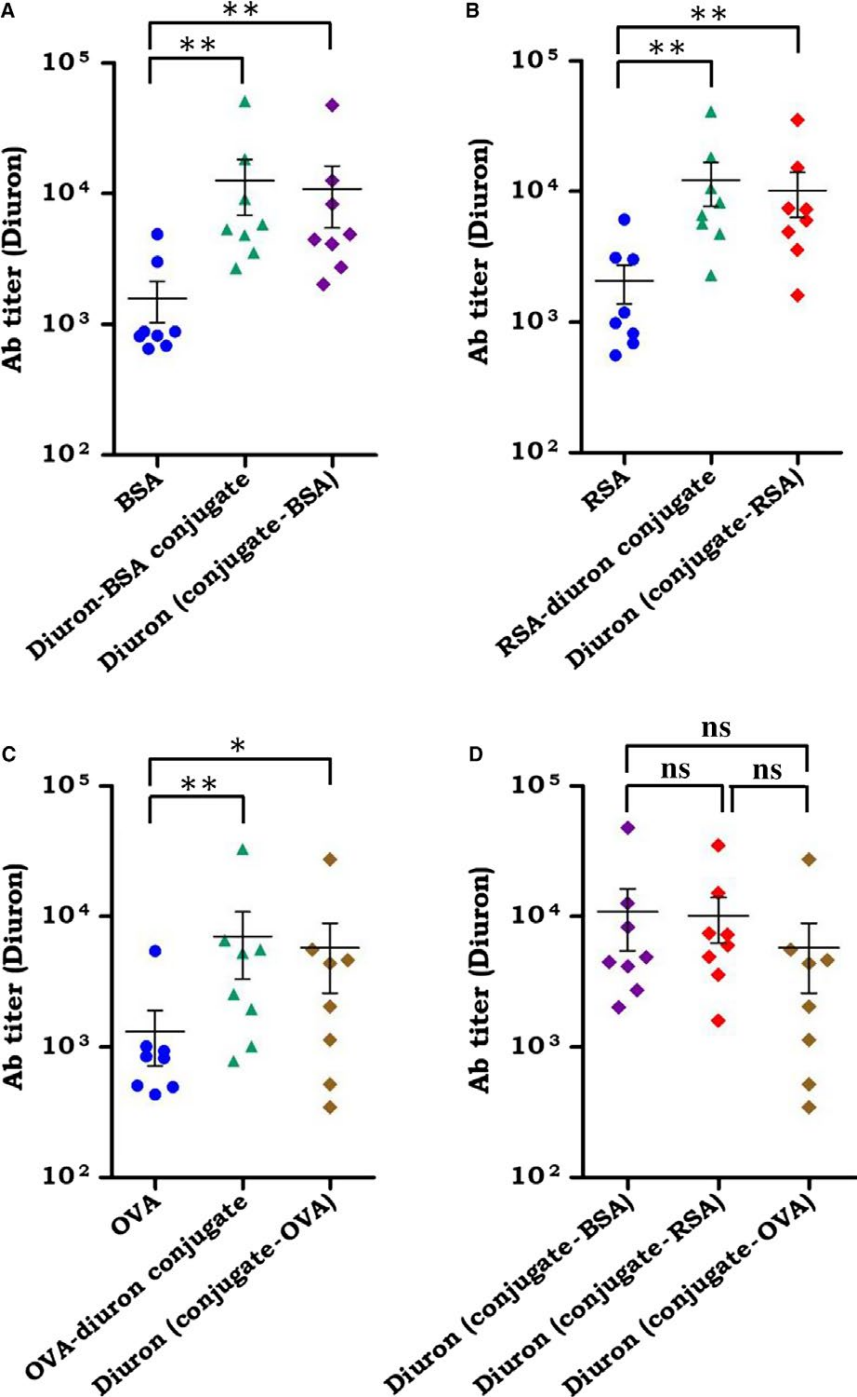
The antibody response detected is specific to diuron. The diuron was conjugated to three different carrier molecules BSA, RSA, and OVA. Antibody titer against diuron was estimated in the serum of 8 individuals that showed high Ab titer by ELISA. (A) Ab titer against BSA, diuron‐BSA conjugate and diuron alone; (B) Ab titer against RSA, diuron‐RSA and diuron alone; (C) Ab titer against OVA, diuron‐OVA, and diuron alone; (D) The average Ab titer (conjugate minus protein) against diuron linked to BSA, RSA, and OVA, respectively, is depicted in the figure. The data represented as mean ± SEM are shown in the form of dot plots. Each dot signifies one individual. *p < 0.05, **p < 0.01

The specificity of serum antibodies against diuron linked with three different carrier molecules BSA, RSA, and OVA were determined. The hapten density of diuron molecules on BSA, RSA, and OVA was 10, 8, and 5, correspondingly. A normalized number (~10) of diuron molecules on carrier protein was calculated. Accordingly, ELISA plates were coated with the three conjugates on the basis of molar concentration. Antibody titer was determined against diuron‐BSA, diuron‐RSA, and diuron‐OVA. The BSA, RSA, and OVA were taken as controls. The sera of the individuals that tested positive for anti‐diuron Abs were selected for the experiment. There was a significant (*p* < 0.01) increase in the Ab titer of all the tested 8 subjects against BSA‐diuron conjugate, as compared to BSA alone. Further, the figure depicts the Ab response against “diuron,” as well (Ab titer of conjugate minus protein). Therefore, suggesting the specificity of Abs against diuron and not BSA (Figure 3A). Similar to BSA, we observed a substantial increase in the Ab titer against RSA‐diuron conjugate (*p < *0.01) and OVA‐diuron conjugate (*p < *0.01), when compared to RSA and OVA alone, respectively (Figure 3B,C). Further, to establish the affinity of Abs against diuron, the average Ab titer (conjugate minus protein) against diuron was observed, that is, 7696, 5907, and 2805, when linked to BSA, RSA, and OVA, respectively (Figure 3D). There was no significant difference in the Ab titer against diuron, when they were coupled with different carrier molecules. This clearly demonstrates that the Ab response was specific against diuron, irrespective of the carrier molecules.

### 3.4 Cancer patients show a significantly lower level of anti‐xenobiotics Abs, as compared to their healthy household contacts

**Figure 4:**
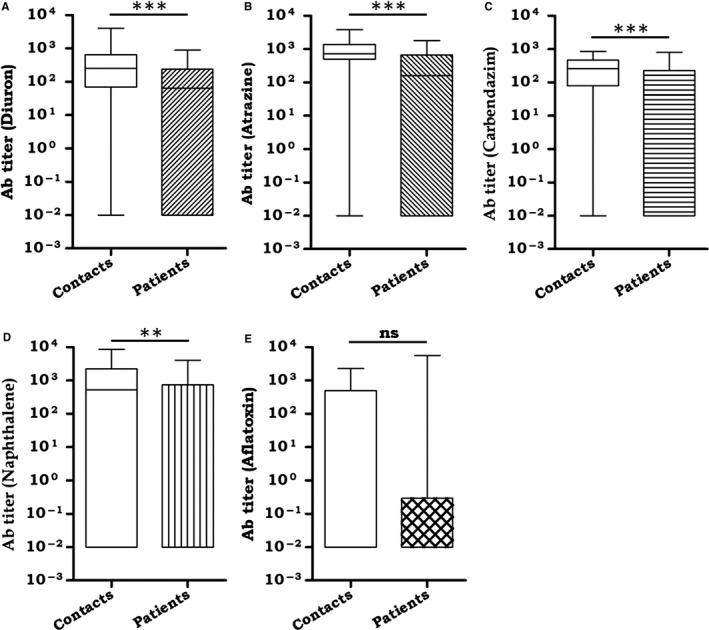
Cancer patients exhibited a significantly lower level of anti‐xenobiotic Abs, as compared to their healthy household contacts. Ab response against xenobiotics was estimated by ELISA in the serum of cancer patients and their healthy household contacts. Ab response was monitored against (A) diuron; (B) atrazine; (C) carbendazim; (D) naphthalene; (E) aflatoxin. The data of the Ab titer against xenobiotics are expressed in the form of box plots. The background Ab titer against the carrier protein BSA was subtracted from the Ab titer of BSA‐xenobiotics. Each box represents the total number of individual (number of contacts: 30, number of patients: 75). **p < 0.01, ***p < 0.001

Serum samples from cancer patients and their healthy household contacts were tested for the presence of anti‐xenobiotic Abs. Diuron, a derivative of phenyl urea, is a herbicide that is widely used in agriculture to control a broad variety of weeds.^19^, ^20^ The United States Environmental Protection Agency (USEPA) classifies diuron as a “known/likely” carcinogen to humans.^21^ Intriguingly, the cancer patients showed significantly (*p* < 0.001) lower Ab titer, as compared to their healthy household contacts (Figure 4A).

Atrazine is a triazine herbicide used worldwide to control the growth of broadleaf weeds. Several investigators have confirmed the contamination of drinking water with atrazine.^22^ The higher level of atrazine was detected in individuals who work in agricultural fields or live nearby, as compared to the urban population.^23^ Epidemiological studies have observed a correlation between atrazine exposure and an increase of premature delivery of babies, birth defects, and induction of cancer.^24^, ^25^, ^26^ The epidemiological studies have shown the role of atrazine in inducing Non‐Hodgkin lymphoma.^27^ Further, it is a high threat for prostate cancer,^28^ as well as ovarian cancer.^29^ Like diuron, the cancer patients showed remarkably (*p* < 0.001) lower Ab titer against atrazine, as compared to their healthy household contacts (Figure 4B).

Carbendazim (methyl‐2‐benzimidazole carbamate) is a broad‐spectrum benzimidazole fungicide used in agricultural industry. It has been shown to induce hepatocarcinoma as well as produces teratogenic effects.^30^, ^31^ Carbendazim can produce aneuploidy by inhibiting the formation of microtubules during cell division.^32^, ^33^ The cancer patients exhibited sufficiently (*p* < 0.001) lower Ab titer against carbendazim, as compared to their healthy household contacts (Figure 4C).

Besides pesticides, we also monitored the Ab response against polycyclic aromatic hydrocarbon, naphthalene. Industrial wastes, biomass burning, tailpipes emission, and cigarettes smoke are the major source of naphthalene causing pollution to the environment. Naphthalene is used as an insect repellent and fumigant.^34^ Naphthalene exposure is linked to various health hazards, including nasal tumors.^35^ We distinguished that cancer patients exhibited significantly (*p* < 0.01) reduced Ab titer against naphthalene, as compared to their healthy household contacts (Figure 4D).

Aflatoxins are the most potent natural carcinogens produced by *Aspergillus* spp. Aflatoxin exposure through crops leads to serious health hazard to humans.^36^ Aflatoxins have carcinogenic properties and toxic effect on the liver.^37^ Among all the natural aflatoxins (B_1_, B_2_, G_1_, and G_2_), aflatoxins‐B1 is a most potent natural carcinogen and induces inflammatory responses.^36^, ^38^ Although we could notice the generation of Abs against aflatoxin in the serum of both patients and healthy individual, there was no statistically significant difference between the two groups (Figure 4E). However, anti‐aflatoxin Abs were absent in majority of the cancer patients. Currently, it is difficult to explain the reason for this discrepancy with other xenobiotics but it appears that the percentage of low Abs level is higher in the patients.

These results signify that the presence of higher level of anti‐xenobiotic Abs in the healthy household contacts may be responsible for neutralizing the toxic effects of xenobiotics. The Abs generated may help in clearing off xenobiotics from the body of healthy individuals, thus protecting them from cancer. On the contrary, the lower occurrence of anti‐xenobiotic Abs in the serum of cancer patients may not be sufficient to eliminate xenobiotics. Hence, these xenobiotics retained in the body may be responsible for triggering cancer.

### 3.5 Highest Ab response was observed against naphthalene in the serum of cancer patients and their healthy household contacts

**Figure 5:**
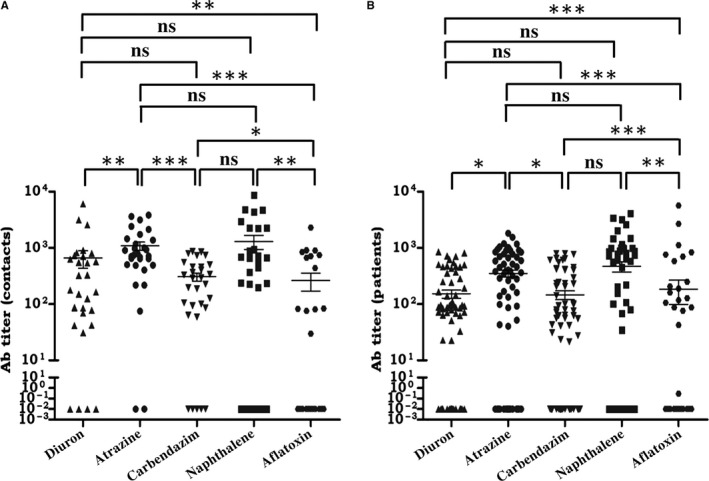
Naphthalene shows maximum Ab titer compared to diuron, atrazine, carbendazim, and aflatoxin. Ab response against diuron, atrazine, carbendazim, naphthalene, and aflatoxin was estimated by ELISA in the serum of (A) healthy individuals; (B) cancer patients. Each dot represents a single individual. The background Ab titer against the carrier protein BSA was subtracted from the Ab titer of BSA‐xenobiotics. *p < 0.05, **p < 0.01,***p < 0.001

We next did a comparative analysis of Ab titers of the diuron, atrazine, carbendazim, naphthalene, and aflatoxin. It was observed that the healthy household contacts showed the highest Ab titer against naphthalene. This difference was significant (*p* < 0.01), as compared to aflatoxin (Figure 5A). After naphthalene, the Ab response against atrazine was also considerably high, when compared with carbendazim (*p* < 0.001), aflatoxin (*p* < 0.001), and diuron (*p* < 0.01). The healthy household contacts also possess high anti‐diuron Abs and anti‐carbendazim Abs, in contrast to anti‐aflatoxin Abs (*p* < 0.01 and *p* < 0.05, respectively). Among the cancer patients, the highest Ab titer was observed against naphthalene, followed by atrazine, aflatoxin, diuron, and least response was observed in the case of carbendazim (Figure 5B).

### 3.6 Breast cancer patients showed lower anti‐xenobiotics Ab titers, as compared to their healthy household contacts

**Figure 6:**
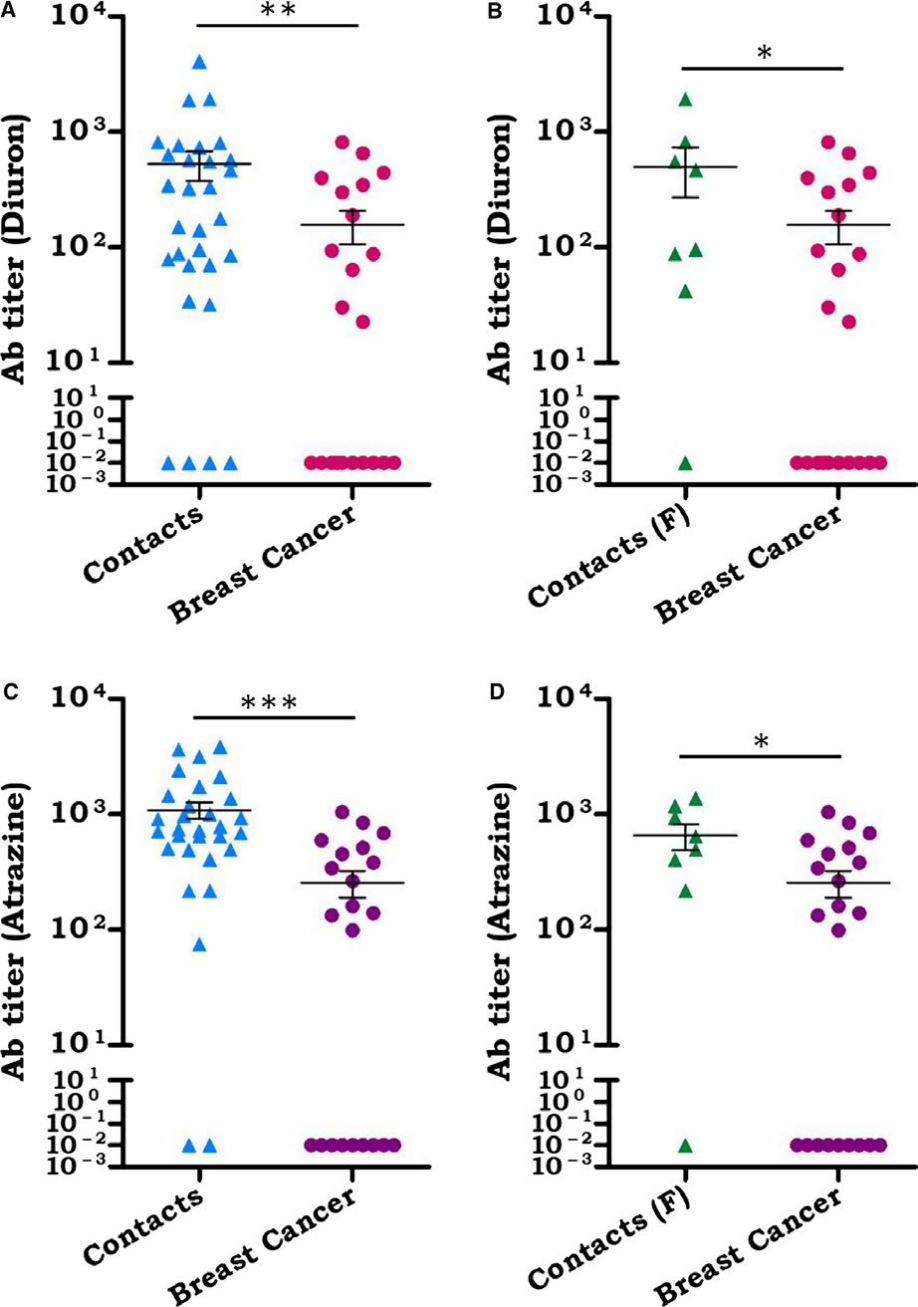
Breast cancer patients exhibited a lower level of anti‐xenobiotic Abs, as compared to their healthy household contacts. Ab response against (A, B) diuron; (C, D) atrazine was estimated by ELISA in the serum of breast cancer patients and healthy household contacts. Comparative analysis of Ab titer against diuron in the serum of (A) breast cancer patients (n: 22) vs healthy household contacts (n: 30); (B) breast cancer patients (n: 22) vs healthy female household contacts (n: 8). Abs response was determined against atrazine in the serum of (C) breast cancer patients (n: 22) vs healthy household contacts (n: 30); (D) breast cancer patients (n: 22) vs sex‐matched healthy household contacts (n: 8). The data shown as mean ± SEM of the Ab titer are expressed in the form of dot plots. Each dot is symbolized as a single individual. *p < 0.05, **p < 0.01,***p < 0.001

The balanced endocrine system is responsible for normal growth and function of the mammary glands. Xenobiotics have been reported to disturb the hormonal homeostasis by interfering with their functions.^39^ Thus, disturbance in the endocrine hormonal balance increases the risk of development of breast cancer. Xenobiotics such as organochlorine pesticides (OCPs) and polychlorinated biphenyls (PCBs) were found to be potent endocrine distressing molecules.^40^, ^41^ The OCPs showed endocrine disturbing property by various mechanisms such as mimicking endogenous hormones, competitive inhibition by binding to endocrine receptors and disturbing metabolism and transport of hormones.^42^ A human study has established a link between OCPs exposure and increased risk of breast cancer.^43^ Therefore, we evaluated the correlation of anti‐xenobiotics Ab and their prevalence with the incidences of breast cancer. We observed a significant (*p* < 0.01) decrease in the Ab titer against diuron in the breast cancer patients, as compared to their healthy household contacts (Figure 6A). Further, female breast cancer patients exhibited considerably (*p* < 0.05) lower Ab titer against diuron, as compared to their healthy household counterparts (Figure 6B). The diminished level of anti‐diuron Ab titer among the cancer patients can be correlated with a higher prevalence of breast cancer in the patients of Punjab province of India.^44^ It may be interpreted from these results that low level of anti‐diuron Abs may be one of the reasons for disease progression in breast cancer patients. However, healthy household contacts remain protected due to the clearance of diuron from the body via high level of anti‐diuron Abs. We also observed a significant (*p* < 0.001) decrease in the Ab titer against atrazine in the breast cancer patients, as compared to their healthy household contacts (Figure 6C). Atrazine has been reported to provoke breast cancer.^45^, ^46^ In addition, breast cancer patients showed lower Ab titer (*p* < 0.05) against atrazine, as compared to their healthy female household contacts (Figure 6D). We hypothesized that the low level of anti‐atrazine Ab in the serum of breast cancer patients could be one of the reasons for the development of breast cancer.

### 3.7 Distinct Ab titer against xenobiotics in cancer patients and their healthy subjects

**Figure 7:**
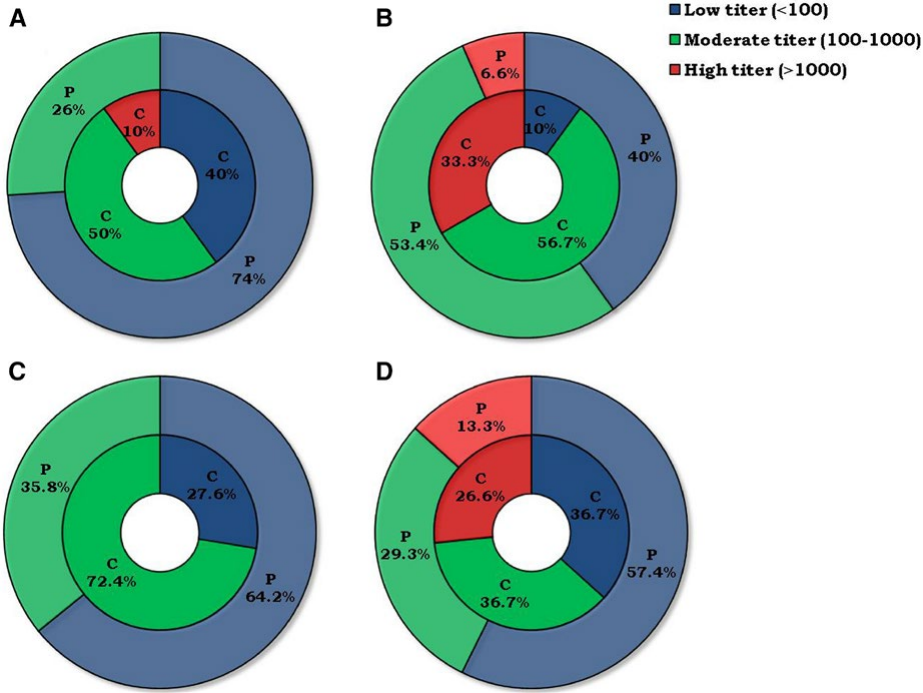
The cancer patients show low Ab titer against xenobiotics. The percentage of individuals was categorized on the basis of three different Ab titer ranges, that is, high (>1000), moderate (100‐1000) and low (<100) for the (A) diuron; (B) atrazine; (C) carbendazim; (D) naphthalene. The percentage was calculated taking into consideration a total number of individuals in each group divided by the number of subjects showing either low or moderate or high Ab titer and represented in a form of double donuts diagram. The numbers of cancer patients involved in the study were 75 and healthy household contacts were 30. “C” indicates controls and “P” patients

We next categorized the patients and healthy individuals on the basis of low (titer <100), moderate (titer 100‐1000) and high (titer >1000) anti‐xenobiotic Ab titers. It is worth to mention here that the percentage of low Ab titer was maximum in the cancer patients, irrespective of the xenobiotics tested. In contrast, it was highest in the healthy household contacts (Figure 7). About 74% of cancer patients demonstrated low anti‐diuron Ab (titer <100), as compared to 40% of household contacts (Figure 7A). About 26% of cancer patients showed moderate anti‐diuron Ab titers (titer 100‐1000), as compared to 50% of household contacts. However, only 10% of household contacts showed high anti‐diuron Ab titer (titer >1000) but could not be detected in the serum of any patient. Correspondingly, 40% of cancer patients displayed low anti‐atrazine Ab titer (titer <100), as compared to only 10% in healthy household contacts (Figure 7B). Whereas 33.3% of household contacts exhibited high anti‐atrazine Ab titer (titer >1000), as compared to cancer patients (6.6%). Further, 64.2% of cancer patients showed lower anti‐carbendazim Ab titer (titer <100), as compared to 27.6% of the household contacts (Figure 7C). About 72.4% of household contacts showed moderate anti‐carbendazim Ab titer (titer 100‐1000) compared to 35.8% of cancer patients (Figure 7C). Furthermore, low level of anti‐naphthalene Abs (titer <100) was detected in the 57.4% of cancer patients, when compared to 36.7% of the household contacts (Figure 7D). About 29.3% of cancer patients showed moderated anti‐naphthalene Ab titer (100‐1000), as compared to 36.7% of household contacts. The 26.6% of the household contacts showed high anti‐naphthalene Abs titer (titer >1000) as compared to cancer patients (13.3%). It may be concluded that the prevalence of high anti‐xenobiotic Abs in healthy household contacts may possibly be one of the rationales for neutralizing the effect of xenobiotics and clearing them from the body through anti‐xenobiotic Abs immune complexes. On the other hand, the low level of anti‐xenobiotics Abs in cancer patients could be a probable factor for induction of cancer.

## 4 DISCUSSION

Regular application of xenobiotics in a particular niche can increase its level in the environment and ultimately exposure to all the organisms present in the vicinity. Majority of xenobiotics are catabolized and excreted out of the body but some show resistance toward metabolism and excretion pathways. Hence, they accumulate in various tissues of the body. Adipose tissues analysis reveals that it contains a diversity of xenobiotics.^47^ The human breast milk was found to be contaminated with the xenobiotics bisphenol‐A, phthalates, polycyclic aromatic hydrocarbons, polychlorinated biphenyls, dichlorophenylchloroethane (DDT), dioxins, organochlorines, organophosphates, and several heavy metals.^48^ Among agricultural workers, xenobiotics exposure was not only found to induce the high risk of cancer but also high mortality rate.^49^

Due to small size, xenobiotics are generally nonimmunogenic. However, it has been reported that some xenobiotics have an ability to directly conjugate with the body proteins.^9^ Consequently, they can elicit an immune response and activate T cells and B cells. Further, nonreactive xenobiotics can be converted to their reactive derivatives by the various xenobiotic metabolism pathways of the host. These reactive haptenic xenobiotics can, as well conjugate with the body proteins. These xenobiotic–protein complexes can act as an immunogen and generate anti‐xenobiotic Ab response. One such example is the production of anti‐drug Abs after long‐term drug therapy. These anti‐drug Abs were found to neutralize the efficacy of the drugs.^12^, ^50^ Detection of anti‐xenobiotic Abs in the serum of agriculture and industrial worker was described as a biomarker for exposure to xenobiotics. Anti‐morphine Abs were detected in the serum of workers involved in narcotics manufacturing.^51^ Similarly, the anti‐benzo(a)pyrene Abs were detected in the serum of children that were dermatologically exposed to the coal tar.^52^

In India, pesticides level has been found to be highest in the Punjab region, as compared to other states. The injudicious use of the pesticides causes contamination of the environment. After the green revolution, a drastic change has been observed in the health of people living in Punjab with a manifold increase in the number of cancer patients.^53^ In 2013, data from Department of Health and Family Welfare indicated that the cancer dominance in the Malwa province of Punjab is highest (1089/million), as compared to India's national average cancer prevalence rate of 800/million. Malwa region is termed as “cancer bowl” of the country. Several studies have correlated the high frequency of cancer in Punjab due to the elevated level of pesticides present in the environmental, as well as human body.^53^, ^54^, ^55^ High rate of DNA damage was observed among the farmers that were occupationally exposed to pesticides in various districts of Punjab.^56^ These genetic defects were observed more in the cotton, paddy, and wheat growers, due to excessive use of pesticides.

In view of the above‐mentioned facts, we investigated the presence of antibodies in the serum of farmers suffering from cancer and their healthy household contacts against xenobiotics diuron, atrazine, carbendazim, naphthalene, and aflatoxin. Further, we correlated the occurrence of Abs against xenobiotics in the serum of cancer patients and their healthy household contacts with the prevention of cancer. Following major findings have emerged from the study: (a) significantly weak Ab titer was noticed in the cancer patients, as compared to healthy subjects; (b) the difference in the Ab level was observed irrespective of the xenobiotics tested; (c) the highest difference was observed in the breast cancer patients; (d) lowest titer of Abs were detected in the cancer patients, contrary to highest Ab concentration among healthy volunteers; (e) the Abs response evaluated was specific to xenobiotics. The presence of the Abs in the serum is an indicator of the exposure to xenobiotics. Xenobiotics have a considerable role in the induction of cancer. Therefore, it was critical to monitor the level of the anti‐xenobiotic Abs in the serum of cancer patients. The elevated level of Abs was predominantly found in the serum of healthy household contacts but to a lesser extent in the cancer patients against pesticides diuron, atrazine, and carbendazim. These pesticides are extensively applied to the crops cultivated in Malwa, Punjab. Thus, agricultural farmers were more susceptible to the exposure of these pesticides due to spraying, careless handling, and inappropriate storage of pesticides.^57^ The constant exposure of these pesticides might led to cancer in the patients. However, elevated levels of Abs in the serum of healthy household contacts can be considered as one of the prominent explanation for being protected from cancer. Even though healthy individuals might have been equally exposed to xenobiotics, as compared to the cancer patients due to equal hours devoted in the agricultural fields, the cancer patients showed significantly lower Ab titers.

Among various cancers, breast cancer patients are highest in the Punjab.^44^ We observed in our study that breast cancer patients exhibited a significant decrease in the anti‐xenobiotic Abs, when compared to their healthy household contacts. Further, similar results were noted in the case of their sex‐matched female healthy household contacts. Atrazine provokes mammary tumors by disrupting endocrine activities in the patients.^58^ Thus, the existence of a low level of anti‐atrazine Abs can be very well correlated with the high prevalence of breast cancer in Punjab. Alternatively, the presence of high level of the anti‐atrazine Abs can play a preventive role against the cancer induction in the household contacts. To date, no such study has demonstrated a link between carbendazim exposures with breast cancer.

Next, we categorized patients and healthy subjects on the basis of low, moderate, and high titers of anti‐xenobiotics Abs in the serum. The majority of cancer patients exhibited low anti‐xenobiotic Abs level. In contrast, the household contacts showed a higher level of anti‐xenobiotics Ab titers. These results further indicate that the increased rate of cancer may be due to a lower level of anti‐xenobiotic Abs among the cancer patients. Further, household contacts may be protected because of the high level of anti‐xenobiotic Abs.

In this study, we have found overlapping Abs response in a few cancer patients and healthy household contacts. Some individuals in household contacts showed a low level of anti‐xenobiotic Abs in their serum. These individuals in household contacts might be at a risk of developing cancer. This requires follow‐up studies and therefore can be an interesting line of future investigation. On the other hand, there are few individuals in the cancer patients that showed high anti‐xenobiotic Ab response in their serum. This may be due to the recovery of patients from cancer after anti‐cancer therapies.

Genetic makeup, as well as environmental factors, contributes to cancer induction. Based on existing results, it is difficult to conclude that higher titer of anti‐xenobiotics Abs may be the sole cause of prevention against cancer. There may be several other factors that may determine the susceptibility to the disease. There are many causes that can contribute to cancer like an infection of some viruses and bacteria, radiations, spontaneous mutations.

We propose that the prevention against exposure to xenobiotics can substantially reduce cancer. Ab‐based therapies can be an admirable option to diminish a possibility of cancer in the individuals that are constantly exposed to the xenobiotics. In many diseases, neutralizing Abs can provide protection against many pathogens, which are responsible for diseases such as measles, diphtheria, botulism, hepatitis, tetanus, rabies, vaccinia, AIDS, etc.^59^, ^60^ More importantly, a strategy employing xenobiotic–carrier complex as a vaccine to immunize individuals living in a high xenobiotic exposure zone can be used to protect them against cancer. Such immunization will elicit anti‐xenobiotic Abs in the individuals. Therefore, any exposure to xenobiotic will be eliminated by the preformed Abs against the xenobiotics.

Based on our observations, we conclude that the prevalence of high level of anti‐xenobiotic Abs in healthy individuals may be one of the causes for protecting them from cancer by neutralizing the effect of xenobiotics present in the body. In contrast, the low titer of Abs may be inefficient in clearing the xenobiotics and therefore these chemicals get adequate opportunity to inflict people with their carcinogenic properties. We suggest that administration of anti‐xenobiotic Abs may be a prudent prophylactic approach to prevent people suffering from xenobiotic‐induced cancer. Furthermore, development of vaccines against xenobiotics can be a cardinal prophylactic measure to considerably control the xenobiotics propagated diseases.

## CONFLICT OF INTEREST

The authors declare that the research was conducted in the absence of any commercial or financial relationships that could be construed as a potential conflict of interest.
