# Supplementary figures and images for: Low prevalence of anti‐xenobiotic antibodies among the occupationally exposed individuals is associated with a high risk of cancer
Source: Cancer Med. 2018 Dec 21;8(1):246–60. doi: 10.1002/cam4.1773 (PMC6346253; doi:10.1002/cam4.1773)

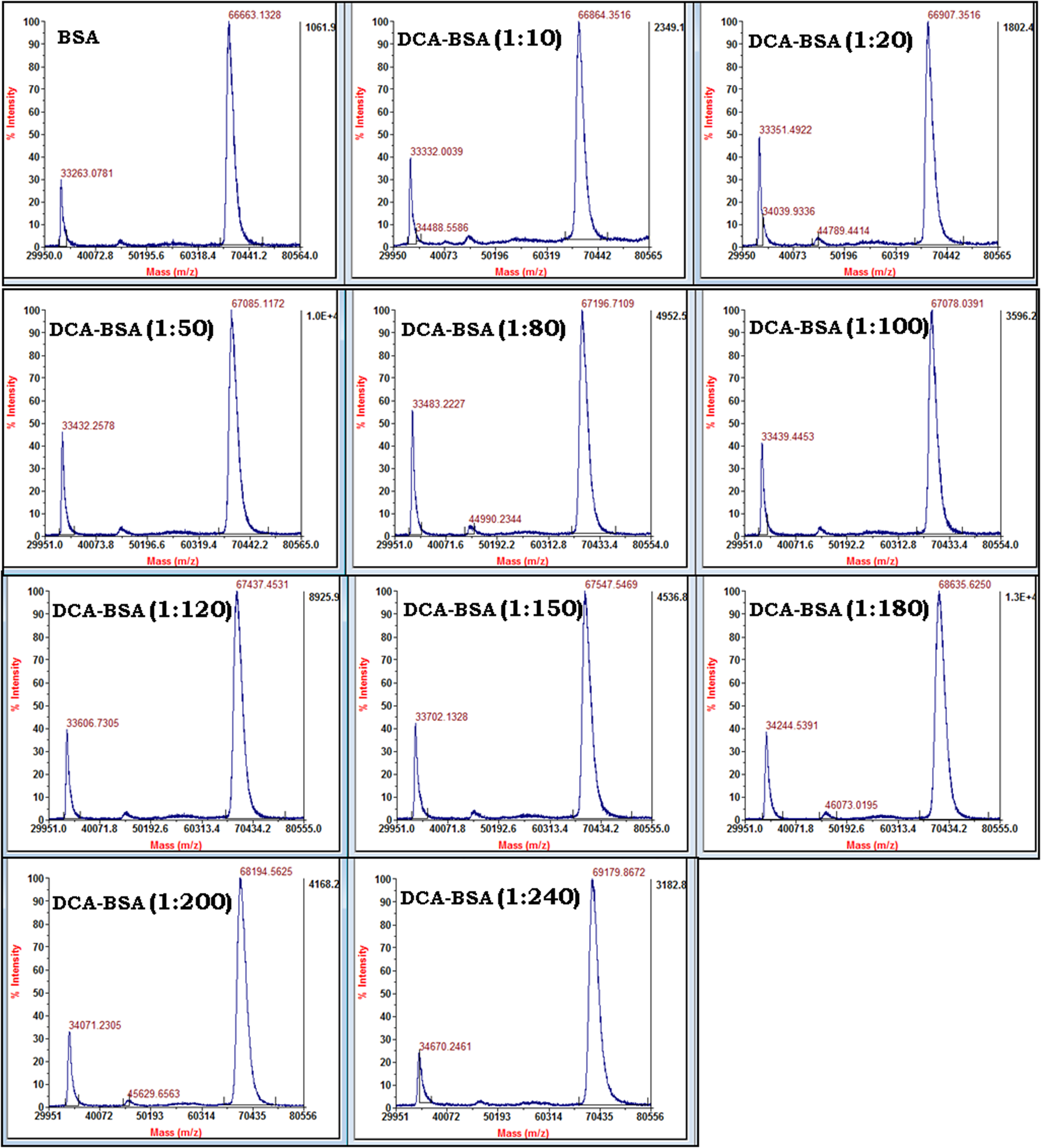

Supplement: Supplementary file 1 [file CAM4-8-246-s001.tif]

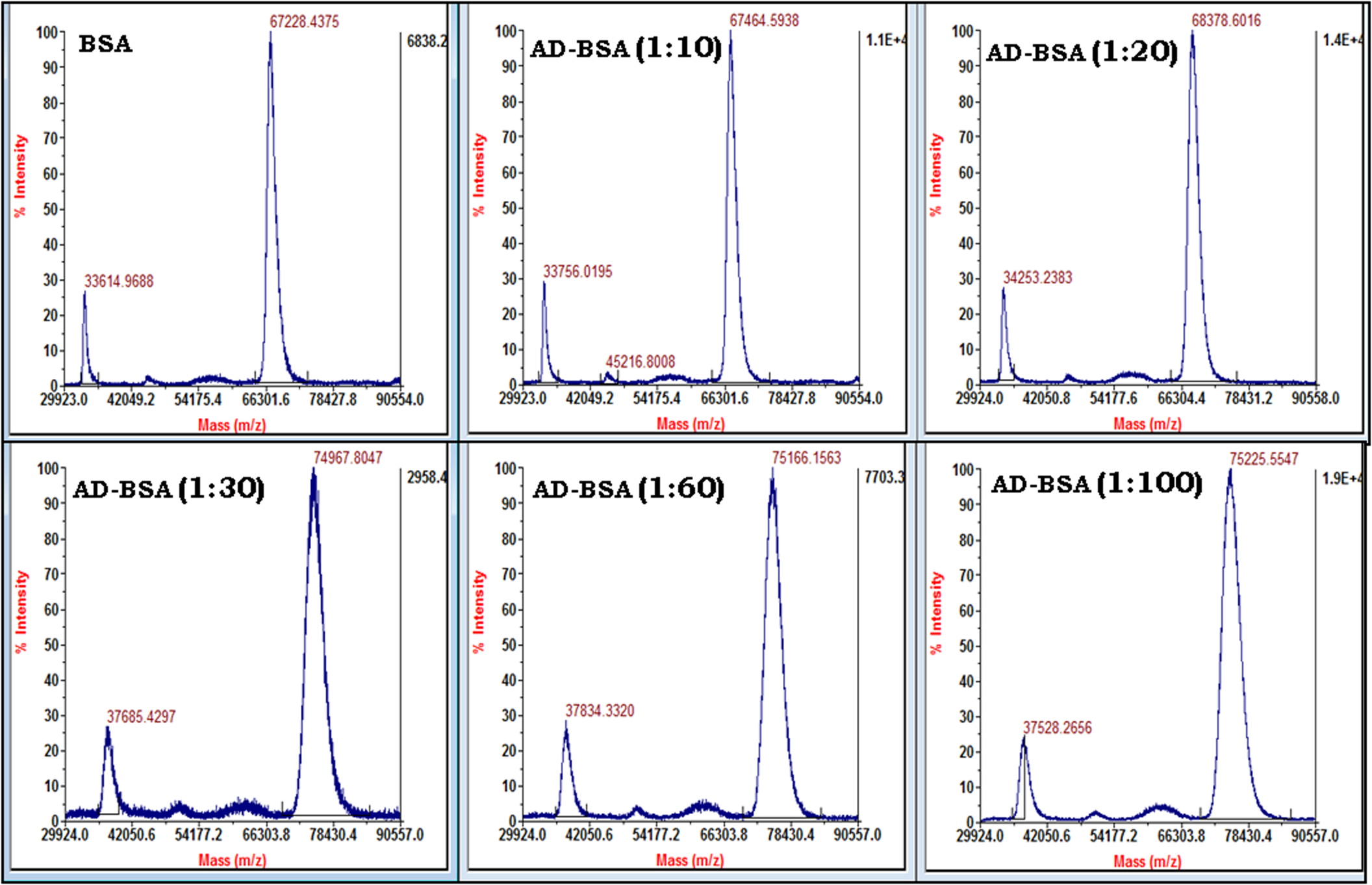

Supplement: Supplementary file 2 [file CAM4-8-246-s002.tif]

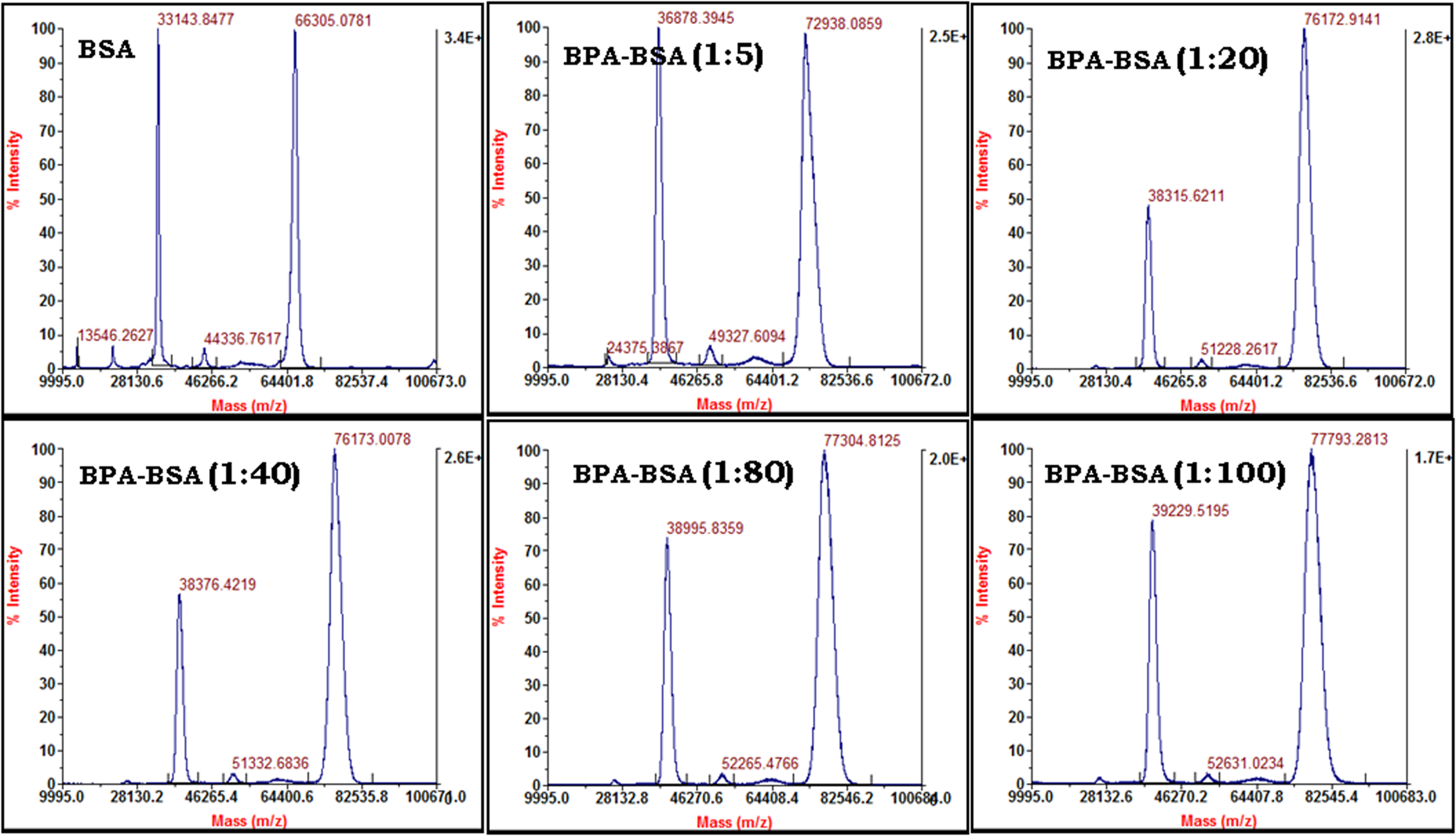

Supplement: Supplementary file 3 [file CAM4-8-246-s003.tif]

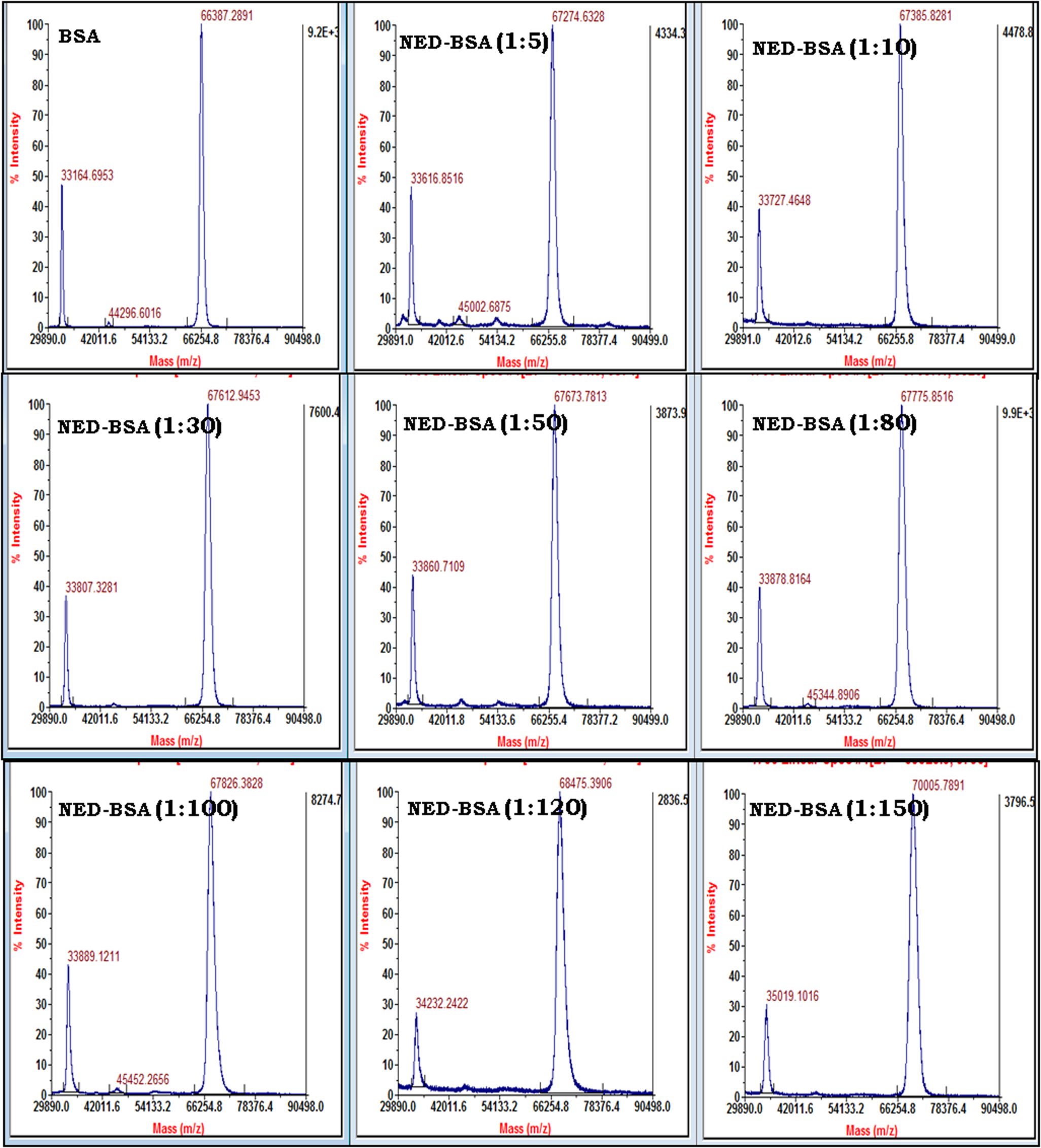

Supplement: Supplementary file 4 [file CAM4-8-246-s004.tif]

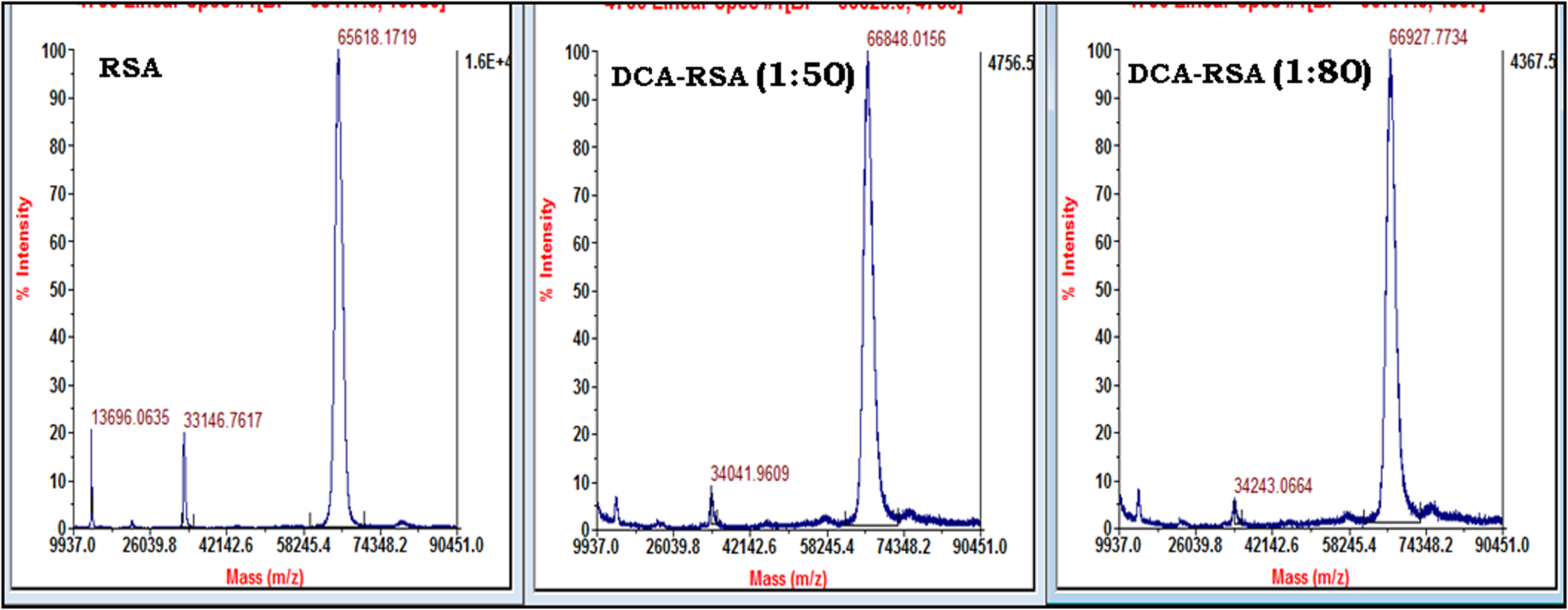

Supplement: Supplementary file 5 [file CAM4-8-246-s005.tif]

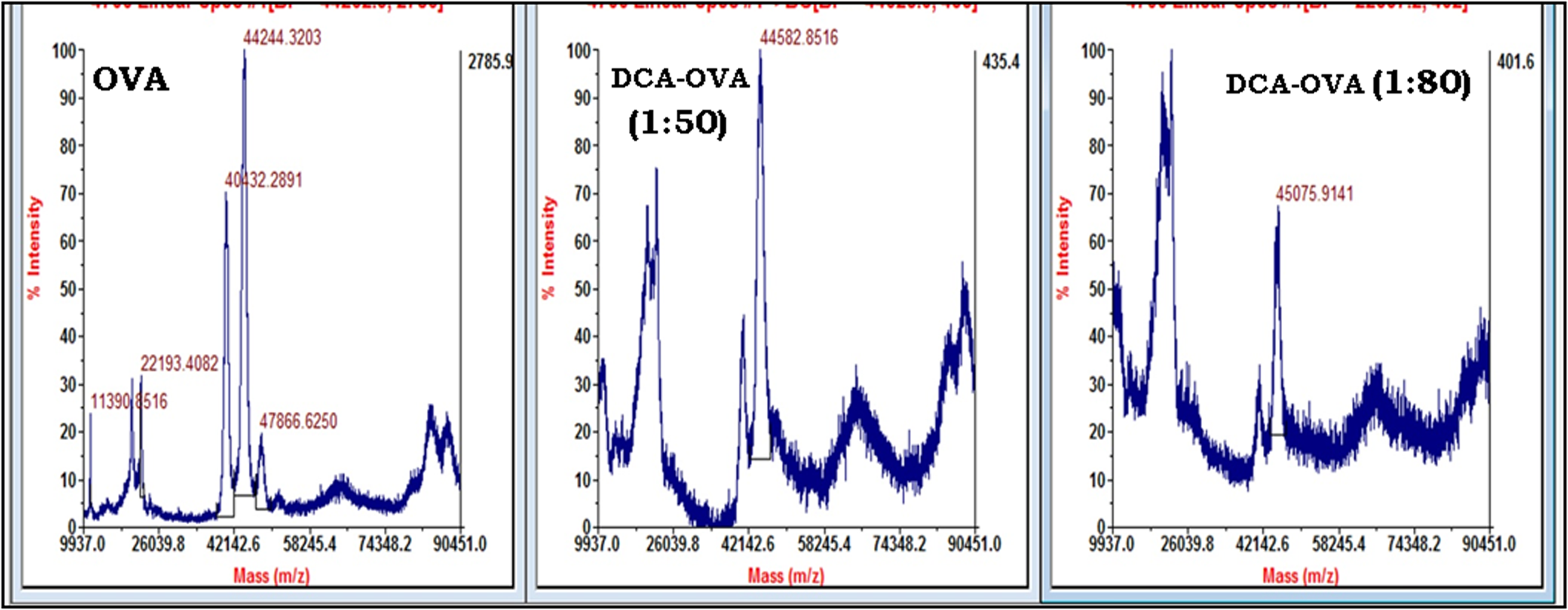

Supplement: Supplementary file 6 [file CAM4-8-246-s006.tif]
