# Supplementary material for: Low prevalence of anti‐xenobiotic antibodies among the occupationally exposed individuals is associated with a high risk of cancer
Source: Cancer Med. 2018 Dec 21;8(1):246–60. doi: 10.1002/cam4.1773 (PMC6346253; doi:10.1002/cam4.1773)
Supplement: Supplementary file 7 [file CAM4-8-246-s007.docx]

**Table 1.** Number of cancer patients and healthy individuals

| **S. No** | **Healthy/Types of Carcinoma** | **Total number of sample** |
| --- | --- | --- |
| 1. | Healthy (household contacts) | 30 |
| 2. | Breast carcinoma | 22 |
| 3. | Ovarian carcinoma | 6 |
| 4. | Cervix carcinoma | 6 |
| 5. | Oesophagus carcinoma | 4 |
| 6. | Lung carcinoma | 4 |
| 7. | Gall bladder carcinoma | 3 |
| 8. | Squamous cell carcinoma | 3 |
| 9. | Tongue carcinoma | 3 |
| 10. | Hodgkin’s lymphoma | 3 |
| 11. | Non-Hodgkin’s lymphoma | 3 |
| 12. | Miscellaneous carcinoma (sample size=1-2) | 18 |
|  | Total number of patients | 75 |

**Table 2.** Mass spectra analysis of dichloroaniline (DCA) conjugated to BSA

| **BSA : DCA ratio** | **Observed mass (D)** | **Change in mass (Δm)** | **Number of DCA molecules/BSA** |
| --- | --- | --- | --- |
| 1:0 | 66663.13 | 0 | 0 |
| 1:10 | 66864.35 | 201.22 | 1.24 (1) |
| 1:20 | 66907.35 | 244.22 | 1.50 (2) |
| 1:50 | 67085.11 | 421.98 | 2.60 (3) |
| 1:80 | 67196.71 | 533.58 | 3.29 (3) |
| 1:100 | 67078.03 | 414.90 | 2.56 (3) |
| 1:120 | 67437.45 | 774.32 | 4.77 (5) |
| 1:150 | 67547.54 | 884.41 | 5.45 (5) |
| 1:180 | 68194.56 | 1531.43 | 9.45 (9) |
| 1:200 | 68635.62 | 1972.49 | 12.17 (12) |
| 1:240 | 69179.86 | 2516.73 | 15.53 (16) |

**Table 2. *Mass spectra analysis of dichloroaniline (DCA) conjugated to BSA.*** The difference in the molecular mass was calculated and number of DCA molecule conjugated to per BSA was determined. It was observed that approximately 16 molecules of DCA were coupled to BSA, at a ratio 1:240.

**Table 3.** Mass spectra analysis of atrazine desethyl (AD) conjugated to BSA

| **BSA : AD**  **ratio** | **Observed**  **mass (D)** | **Change in**  **mass (Δm)** | **Number of AD molecule/BSA** |
| --- | --- | --- | --- |
| 1:0 | 66842.89 | 0 | 0 |
| 1:10 | 67464.59 | 621.70 | 3.31 (3) |
| 1:40 | 68378.60 | 1535.71 | 8.18 (8) |
| 1:30 | 74963.62 | 8120.73 | 43.28 (43) |
| 1:60 | 75162.04 | 8319.15 | 44.33 (44) |
| 1:100 | 75225.55 | 8382.66 | 44.67 (45) |

**Table 3. *Higher ratio of AD molecules to BSA leads to increased conjugation of AD molecules to per BSA*.** The difference between the molecular mass of conjugate and BSA, exhibited that maximum number of conjugation of AD (~43) to BSA at ratio of 1:100.

**Table 4.** Mass spectra analysis of benzimidazole propionic acid (BPA) conjugated to BSA

| **BSA : BPA ratio** | **Observed**  **mass (D)** | **Change in mass (Δm)** | **Number of BPA molecule/BSA** |
| --- | --- | --- | --- |
| 1:0 | 66305.07 | 0 | 0 |
| 1:5 | 72938.08 | 6633.01 | 34.87 |
| 1:20 | 76172.91 | 9867.84 | 51.88 |
| 1:40 | 76173.00 | 9867.93 | 51.88 |
| 1:80 | 77304.81 | 10999.74 | 57.83 |
| 1:100 | 77793.28 | 11488.21 | 60.40 |

**Table 4. *Increased molar ratio of BPA for conjugation with BSA leads to increased molecular mass of BSA*.** The change in mass of conjugate and BSA showed maximum number BPA (~60) attached to BSA at ratio of 1:100.

**Table 5.** Mass spectra analysis of N-(1-naphthyl)-ethylenediamine (NED) conjugated to BSA

| **BSA : NED**  **ratio** | **Observed mass (D)** | **Change in mass (Δm)** | **Number of NED molecule/BSA** |
| --- | --- | --- | --- |
| 1:0 | 66387.28 | 0 | 0 |
| 1:5 | 67274.63 | 887.35 | 3.42 (3) |
| 1:10 | 67385.82 | 998.54 | 3.85 (4) |
| 1:30 | 67612.94 | 1225.66 | 4.73 (5) |
| 1:50 | 67673.78 | 1286.50 | 4.96 (5) |
| 1:80 | 67775.85 | 1388.57 | 5.35 (5) |
| 1:100 | 67826.38 | 1439.10 | 5.55 (6) |
| 1:120 | 68475.39 | 2088.11 | 8.05 (8) |
| 1:150 | 70005.78 | 3618.50 | 13.93 (14) |

**Table 5. *Elevated molar ratio of NED to BSA leads to increased conjugation of NED molecules to each BSA*.** The change in the molecular mass between conjugates and BSA exhibited that maximum 14 molecules of NED were linked to BSA, at ratio of 1:150.

**Table 6.** Mass spectra analysis of dichloroaniline (DCA) conjugated to RSA

| **RSA : DCA ratio** | **Observed mass (D)** | **Change in mass (Δm)** | **Number of DCA molecules/RSA** |
| --- | --- | --- | --- |
| 1:0 | 65618.17 | 0 | 0 |
| 1:50 | 66848.01 | 1229.84 | 7.59 (8) |
| 1:80 | 66927.77 | 1309.60 | 8.08 (8) |

**Table 6. *Mass spectra analysis of dichloroaniline (DCA) conjugated to RSA.*** The difference in the molecular mass was determined and number of DCA molecule attached to per RSA was calculated. It was observed that roughly 8 molecules of DCA were coupled to RSA, at a ratio 1:80.

**Table 7.** Mass spectra analysis of dichloroaniline (DCA) conjugated to OVA

| **OVA : DCA ratio** | **Observed mass (D)** | **Change in mass (Δm)** | **Number of DCA molecule/OVA** |
| --- | --- | --- | --- |
| 1:0 | 44244.32 | 0 | 0 |
| 1:50 | 44582.17 | 337.85 | 2.0 (2) |
| 1:80 | 45075.91 | 831.59 | 5.13 (5) |

**Table 7. *Mass spectra analysis of dichloroaniline (DCA) conjugated to OVA.*** The difference in the molecular mass was estimated and number of DCA molecule attached to per OVA was calculated. It was observed that around 5 molecules of DCA were coupled to OVA, at a ratio 1:80.
